# Differences in training among prehospital emergency physicians in Germany

**DOI:** 10.1007/s10049-022-01021-z

**Published:** 2022-04-08

**Authors:** Matthias Bollinger, C. Mathee, A. D. Shapeton, S. C. Thal, S. G. Russo

**Affiliations:** 1 Department of Anesthesiology, Intensive Care, Emergency Medicine and Pain Therapy, Schwarzwald-Baar Hospital, Klinikstr. 11, 78052 Villingen-Schwenningen, Germany; 2 Faculty of Health, School of Medicine, Witten/Herdecke University, Witten, Germany; 3 Helios University Hospital Wuppertal, Witten/Herdecke University, Wuppertal, Germany; 4 Faculty of Medicine, University Medical Center Goettingen, Goettingen, Germany; 5 Boston Veterans Affairs Healthcare System, Boston, MA USA; 6 Tufts University School of Medicine, Boston, MA USA

**Keywords:** Learning curves, Invasive procedures, Education, Emergency medicine, Emergency medical services, Lernkurven, Invasive Maßnahmen, Ausbildung, Notfallmedizin, Rettungsdienst

## Abstract

**Background:**

Germany has an interdisciplinary physician-based emergency medical service. Differences in training likely lead to different levels of expertise.

**Objectives:**

We assessed the number of manual procedures performed at the completion of training to determine level of experience of prehospital emergency physicians of different primary specialties.

**Materials and methods:**

Immediately after passing the board examination each examinee was asked to estimate the number of performed procedures for 26 manual skills. We compared the results with recommendations and data on learning manual skills. Results are presented as mean (standard deviation).

**Results:**

Endotracheal intubation via direct laryngoscopy was performed 1032 (739) times by anesthesiologists. Surgeons and internists performed 89 (89) and 77 (65) intubations, respectively. Intubation via video laryngoscopy was performed 79 (81) times by anesthesiologists, 11 (17) times by surgeons and 6 (11) times by internists. Surgeons had little experience in non-invasive ventilation, with 9 (19) performed procedures and had rarely used external pacemaker therapy or electrical cardioversion. In comparison, among all participants non-invasive ventilation was performed 152 (197) times, electrical cardioversion was performed 41 (103) times and an external pacemaker was used 6 (15) times. For other procedures the numbers did not markedly differ between the different specialties.

**Conclusion:**

The number of performed procedures markedly differed for some skills between different primary specialties. Recommendations regarding a procedural volume were not always met, suggesting missing expertise for some skills. A defined number of procedures should therefore be a formal requirement to be eligible for board certification in prehospital emergency medicine.

## Abstract (de)

**Hintergrund:**

Der Notarztdienst in Deutschland ist interdisziplinär. Es ist wahrscheinlich, dass Unterschiede in der Ausbildung von Notärzten zu Unterschieden in deren Expertise führen.

**Ziel der Arbeit:**

Wir evaluierten die Anzahl durchgeführter manueller Maßnahmen am Ende der Ausbildung, um Unterschiede in der Erfahrung von Notärzten verschiedener Disziplinen festzustellen.

**Material und Methode:**

Unmittelbar nach bestandener Prüfung zur Zusatzbezeichnung Notfallmedizin baten wir Teilnehmende, die Anzahl der Durchführungen für 26 verschiedene manuelle Maßnahmen zu schätzen. Wir verglichen die Ergebnisse mit Empfehlungen und Daten zum Erlernen manueller Fähigkeiten. Die Ergebnisse werden als Mittelwert (Standardabweichung) präsentiert.

**Ergebnisse:**

Die endotracheale Intubation mittels konventioneller Laryngoskopie wurde von Anästhesisten 1032-mal (739) durchgeführt, Chirurgen und Internisten führten 89 (89) bzw. 77 (65) Intubationen durch. Die Intubation mittels Videolaryngoskop wurde von Anästhesisten 79-mal (81) durchgeführt, 11-mal (17) von Chirurgen und 6-mal (11) von Internisten. Chirurgen hatten mit 9 (19) Anwendungen wenig Erfahrung mit nichtinvasiver Beatmung und hatten selten Erfahrung mit der externen Schrittmachertherapie oder der elektrischen Kardioversion sammeln können. Im Vergleich unter allen Teilnehmenden wurde eine nichtinvasive Beatmung 152-mal (197) durchgeführt, die elektrische Kardioversion 41-mal (103) und die externe Schrittmachertherapie 6‑mal (15). Für andere Maßnahmen waren die Unterschiede gering ausgeprägt.

**Schlussfolgerung:**

Die Anzahl der Anwendungen unterschied sich für einige Maßnahmen deutlich zwischen den verschiedenen Disziplinen. Empfehlungen zu Mindestanzahlen an Anwendungen wurden nicht immer befolgt. Eine definierte Anzahl von Anwendungen sollte zukünftig Bestandteil der Weiterbildungsordnung für die Zusatzbezeichnung Notfallmedizin sein.

## Introduction and background

In recent years, prehospital emergency medicine has become an increasingly recognized subspecialty, even in some countries with traditionally “paramedic-based” emergency medical systems. Emergency physicians and anesthesiologists play a major role in this field of medicine.

In Germany—with its “physician-based” emergency medical services—the history of prehospital emergency medicine started in the late 1950s. However, it was not until 1994 that formal training for prehospital emergency physicians was introduced by the German Medical Association (Bundesärztekammer). In 2003 the German Medical Association revised the curriculum and introduced the subspecialty “Emergency Medicine” (Zusatzbezeichung Notfallmedizin) as a qualification for prehospital emergency physicians [1]. The German Medical Association publishes a model curriculum for subspecialty training. Prehospital emergency physicians must have a minimum of 2 years of clinical experience, with a minimum of 6 months in an emergency department, an intensive care unit or in anesthesiology. Additionally, applicants need to complete an 80 h course in emergency medicine and attend 50 out-of-hospital emergency calls under supervision [2]. The model curriculum of the German Medical Association also includes the performance of 5 reductions of subluxations or fractures and performance of 50 endotracheal intubations including the use of video laryngoscopy. However, the responsible authorities for subspecialty certification are the State Chambers of Physicians. These issue their own curriculum, which can differ from the model curriculum of the German Medical Association. The State Chamber of Physicians of North Rhine does not require a number of performed procedures [2]. Formal training is concluded by an oral board exam at the State Chamber of Physicians (Landesärztekammer).

In the past, the German Medical Association incorporated minimum requirements for procedures in their model curriculums. But, in recent years both the German Medical Association and the State Chambers of Physicians have moved away from this approach. Learning outcomes are now operationalized around competencies [3]. The proponents of this approach argue that the required number of a given procedure to gain a competency can vary from physician to physician, and that assessing the number of performed procedures as a quantitative measure does not necessarily provide a qualitative measure of competency [3, 4]. The required competencies are therefore organized in a framework of different levels of expertise. Applicants for board certification have to provide a certificate from their educators that they possess these required competencies.

This approach may be reasonable, given that for most of the skills in this investigation there is insufficient data for clear recommendations regarding a minimum number of procedures. Furthermore, the performance of a recommended number of procedures does not guarantee competence. However, for some skills, e.g., endotracheal intubation, there is high-quality evidence demonstrating procedural volume required to reach a defined learning outcome, as well as established learning curves for this skill [5–10]. Despite these caveats, it is evident that with progressively more experience comes a higher likelihood for skill mastery, regardless of individual talent or learning curve.

Recommendations regarding a minimum of procedures have the advantage of being objectively comparable and do not depend on an educator’s judgement [11]. These recommendations do not necessarily contradict competency-based models and can help learners to self-assess their competencies as knowledge develops with more performed procedures [12]. As a minimum requirement they can help to ensure a minimal expertise with these skills. Ultimately, the importance of such recommendations relies on their evidence, and for the vast majority of manual skills in emergency medicine, high-quality data to guide competencies are lacking.

Due to the long history of prehospital emergency medicine in Germany—with its pioneers from different fields of medicine, predominantly surgery and anesthesiology—the subspecialty is one of the few remaining interdisciplinary subspecialties. This stands in contrast to many other countries where entry into this subspecialty is restricted to physicians of only a few specialties. Previous studies demonstrated that most prehospital emergency physicians in Germany have a background in anesthesiology, followed by physicians from internal medicine, surgery and trauma surgery, limiting the participating specialties in practice [13].

However, differences in training likely lead to different levels of expertise, especially when considering manual skills. Since there are currently no requirements regarding a minimum procedural volume, it is unclear if those undergoing board certification have sufficient experience in critical manual skills necessary for the practice of prehospital emergency medicine.

We conducted a study to evaluate how often emergency physicians applying for board examination performed a variety of procedures in order to determine differences in training between prehospital emergency physicians from different primary specialties. We compared the results with existing recommendations and studies on learning manual skills where available.

## Study design and investigation methods

The study was conducted between July 2019 and September 2020 at the state chamber of physicians of North Rhine after approval of their ethics committee. We initially intended to evaluate all applicants for board exam over a period of 1 year. Due to the coronavirus disease 2019 (COVID-19) pandemic some examinations had been canceled and we therefore decided to extend the survey period. Immediately after passing the board examination each examinee was offered a survey form and asked to participate in the study. The participants were asked 12 questions about demographics, education and their scope of practice. Participants were then asked to estimate the number of performed procedures for 26 different manual skills in both in-hospital and out-of-hospital settings. Since there is no formal requirement of a minimum of performed procedures for board eligibility, it is not possible to collect other than estimated, self-reported data in a survey form.

The data were collected in form of a paper-based anonymous survey. The results were transferred into a spreadsheet in Excel (Excel, Microsoft Corporation, Redmond, WA, USA) and statistical analysis was done with SOFA (Paton-Simpson and Associates Ltd, USA). The respondents’ estimates are presented as mean (standard deviation).

A comprehensive literature review was performed to evaluate whether there is reliable data for learning curves or a procedural volume for skill acquisition of the 26 different skills subject to this investigation. We searched pubmed.gov (National Center for Biotechnology Information, U.S. National Library of Medicine, Rockville Pike, Bethesda MD, USA) for the different skills in conjunction with the keywords “learning” and “skill”. We further screened these results for studies that provide data regarding a procedural volume necessary for skill acquisition. As the value of mannequin studies in this context is unclear, we excluded these studies from further evaluation. The results of this review were listed in tables and compared with the estimated number of performed procedures of the respondents.

## Results

Of 223 forms offered, 113 answered forms were returned (50.67% response rate). One form was excluded due to largely incomplete responses.

Of the 112 participants who were included for further evaluation, 55 were female, 54 male, and 3 did not disclose their gender. The mean age of the participants was 33.5 (5) years (111/112). While 85 participants had not concluded training in a primary specialty, 24 physicians had previously completed training in at least one primary specialty. Anesthesiologists represented the largest group of participants with 55 physicians, followed by internal medicine with 43 participants, surgery with 10 participants and general medicine with 2 participants (110/112).

The mean clinical experience of the participants was 5.9 (2.7) years. In all, 75 participants reported working routinely in intensive care settings (111/112), but only 4 participants reported no previous experience in intensive care (106/112). Participants had a mean experience of 13 (9.3) months in intensive care (106/112). Experience working in an emergency department was reported by 50 participants (111/112) with a mean experience of 9 (13.7) months (107/112). Only 33 participants routinely managed pediatric patients (111/112).

The number of performed procedures markedly differed for some skills between anesthesiologists, surgeons and internists. We decided to present the results for these specialties separately, as well as for all participants combined.

Most of the procedures had been performed far more often in-hospital than out-of-hospital by the participants. We therefore decided to report the number of out-of-hospital performed procedures only where these procedures were performed more frequently than in-hospital.

The results for the estimated number of performed procedures related to airway management are shown in Table 1.

**Table 1:** Airway procedures

Airway procedures	Anesthesiologists (55/112)		Surgeons (10/112)		Internists (45/112)		Total (112)	
	Mean (SD)	*n*	Mean (SD)	*n*	Mean (SD)	*n*	Mean (SD)	*n*
Direct laryngoscopy	1031.7 (738.8)	52/55	89 (88.9)	10/10	76.9 (64.9)	45/45	536.8 (685.0)	109/112
Video laryngoscopy	79.0 (80.7)	53/55	11 (17.1)	10/10	6.1 (11.0)	44/45	42.3 (66.7)	109/112
Laryngeal tube in-hospital	5.5 (19.3)	52/55	2.5 (4.9)	10/10	5.2 (16.0)	45/45	5.0 (16.9)	109/112
Laryngeal tube out-of-hospital	2.2 (4.4)	54/55	3.4 (4.5)	10/10	5.4 (11.7)	44/45	3.6 (8.2)	110/112
Laryngeal mask	597.7 (554.3)	52/55	72.8 (188.0)	9/10	15.0 (28.5)	45/45	304.7 (475.9)	108/112
Cricothyroidotomy	3.1 (6.0)	55/55	2.8 (4.7)	10/10	0.5 (1.6)	45/45	1.9 (4.7)	112/112

Note that numbers of out-of-hospital procedures were only reported where these procedures were performed more frequently than in-hospital. Where not declared, the numbers of in-hospital procedures are reported

*SD* standard deviation

Table 2 shows the results for procedures related to “breathing”.

**Table 2:** Breathing/ventilation strategies

Breathing/ventilation strategies	Anesthesiologists (55/112)		Surgeons (10/112)		Internists (45/112)		Total (112)	
	Mean (SD)	*n*	Mean (SD)	*n*	Mean (SD)	*n*	Mean (SD)	*n*
Bag-valve-mask ventilation	1481.7 (1434.9)	52/55	189.2 (269.3)	9/10	113 (90.8)	45/45	781.2 (1191.9)	108/112
Non-invasive ventilation in-hospital	183.6 (243.7)	53/55	7.3 (12.4)	8/10	142.5 (135.4)	44/45	152.0 (196.6)	107/112
Non-invasive ventilation out-of-hospital	14.6 (23.3)	54/55	8.8 (18.5)	9/10	14.8 (27.1)	44/45	14.0 (24.3)	109/112
Mechanical ventilation	1635.7 (1721.2)	49/55	170.6 (258.2)	9/10	233.1 (255.0)	40/45	919.3 (1370.4)	99/112

Note that numbers of out-of-hospital procedures were only reported where these procedures were performed more frequently than in-hospital. Where not declared the numbers of in-hospital procedures are reported

*SD* standard deviation

Table 3 shows the results for procedures related to “circulation”.

**Table 3:** Circulation procedures

Circulation procedures	Anesthesiologists (55/112)		Surgeons (10/112)		Internists (45/112)		Total (112)	
	Mean (SD)	*n*	Mean (SD)	*n*	Mean (SD)	*n*	Mean (SD)	*n*
Intravenous access	2315.6 (2400.6)	48/55	1057.8 (595.1)	9/10	2033.7 (2615.3)	43/45	2050.2 (2380.5)	102/112
Intraosseous access in-hospital	1.3 (3.3)	54/55	1.5 (3.4)	10/10	6.4 (20.7)	45/45	3.4 (13.5)	111/112
Intraosseous access out-of-hospital	2.7 (4.0)	55/55	6.2 (8.8)	10/10	5.1 (10.0)	44/45	3.9 (7.4)	111/112
Needle thoracocentesis Monaldi position	0.8 (2.0)	52/55	2.7 (4.1)	10/10	2.4 (4.7)	45/45	1.7 (3.7)	109/112
Tube thoracostomy	11.4 (17.4)	54/55	25.4 (31.3)	10/10	7.2 (10.8)	45/45	11.0 (17.3)	111/112
Electrical cardioversion	18.1 (30.0)	55/55	1.4 (3.1)	10/10	80.7 (152.5)	43/45	40.7 (103.0)	110/112
Pacemaker in-hospital	2.3 (6.6)	55/55	0.2 (0.6)	10/10	10.7 (22.3)	45/45	5.5 (15.4)	112/112
Pacemaker out-of-hospital	0.3 (0.8)	55/55	0.3 (0.7)	10/10	0.7 (1.8)	44/45	0.8 (103.0)	110/112
Defibrillation in-hospital	27.2 (83.6)	55/55	7.5 (8.8)	10/10	80.1 (204.1)	43/45	45.9 (143.6)	110/112
Defibrillation out-of-hospital	11.34 (28.0)	53/55	21.5 (45.6)	10/10	8.1 (13.76)	42/45	10.9 (25.3)	107/112
Tourniquet in-hospital	0.02 (0.1)	55/55	0.2 (0.4)	10/10	0 (0)	45/45	0.03 (0.2)	112/112
Tourniquet out-of-hospital	0.3 (0.8)	55/55	0.6 (0.8)	10/10	0.1 (0.3)	44/45	0.2 (0.6)	111/112
Pelvic binder in-hospital	0.1 (0.6)	55/55	2.6 (4.2)	10/10	0.2 (0.8)	45/45	0.7 (3.7)	112/112
Pelvic binder out-of-hospital	2.7 (5.2)	55/55	6.2 (15.5)	10/10	1.8 (2.9)	44/45	2.7 (6.1)	111/112

Note that numbers of out-of-hospital procedures were only reported where these procedures were performed more frequently than in-hospital. Where not declared the numbers of in-hospital procedures are reported

*SD* standard deviation

Ultrasound procedures are reported in Table 4.

**Table 4:** Ultrasound procedures

Ultrasound procedures	Anesthesiologists (55/112)		Surgeons (10/112)		Internists (45/112)		Total (112)	
	Mean (SD)	*n*	Mean (SD)	*n*	Mean (SD)	*n*	Mean (SD)	*n*
FAST ultrasound	39.5 (137.2)	55/55	343.3 (395.6)	9/10	133.2 (195.0)	42/45	102.1 (207.4)	108/112
Lung ultrasound	48.8 (134.7)	55/55	26.9 (62.5)	9/10	117.2 (210.7)	43/45	73.4 (167.1)	109/112
TTE ultrasound	50.6 (151.1)	54/55	103.5 (315.0)	10/10	522.7 (1496.4)	44/45	243.3 (978.4)	110/112

*FAST* Focused Assessment with Sonography in Trauma, *TTE* transthoracic echocardiogram, *SD* standard deviation

The results of the comprehensive literature review are reported in Table 5.

**Table 5:** Studies on procedural volume for learning manual skills and defined outcome parameters. Results of the comprehensive literature review

Skill	Reference	Required procedural volume	Defined outcome parameter
Endotracheal intubation using direct laryngoscopy	Konrad et al. [5]	57	90% Success
	Buis et al. [6]	50	90% Success
	Mulcaster et al. [7]	47	90% Success
	Komatsu et al. [8]	29	80% Success
	Oliveira et al. [9]	43 ± 33.49	80% Success
	Bernhard et al. [10]	150	95% Success
Endotracheal intubation using video laryngoscopy	No applicable studies found		
Laryngeal tube	No applicable studies found		
Laryngeal mask	Mohr et al. [15]	40	86% Success
Cricothyroidotomy	No applicable studies found		
Bag-valve-mask ventilation	Komatsu et al. [8]	27	80% Success
Non-invasive ventilation	No applicable studies found		
Mechanical ventilation	No applicable studies found		
Intravenous access	Oliveira et al. [9]	56 ± 43	80% Success
Intraosseous access	No applicable studies found		
Needle thoracocentesis	No applicable studies found		
Tube thoracostomy	No applicable studies found		
Electrical cardioversion	No applicable studies found		
Pacemaker therapy	No applicable studies found		
Defibrillation	No applicable studies found		
Tourniquet	No applicable studies found		
Pelvic binder	No applicable studies found		
FAST	Ma et al. [27]	35	86.1% Accuracy
Lung ultrasound	No applicable studies found		
TTE ultrasound	Chisholm et al. [29]	45	90% Success in written test

*FAST* Focused Assessment with Sonography in Trauma, TTE transthoracic echocardiogram

## Discussion

The main findings were

- Most physicians applying for board exam in (prehospital) “emergency medicine” had a background in anesthesiology, followed by internal medicine, surgery and general medicine and were in an advanced stage of their primary specialty residency.
- Experience in manual skills differed among different primary specialties. This was especially prominent for airway management skills.
- Only anesthesiologist emergency physicians met the recommended minimum number of procedures for endotracheal intubation.
- While surgeons had more experience in performing tube thoracostomies, this was not true for cricothyroidotomy, which was performed rarely by all respondents.
- Surgeons had very little experience with non-invasive ventilation, pacemaker therapy and electrical cardioversion.

In this study we evaluated the level of experience of German prehospital emergency physicians at the time of their board certification at the State Chamber of Physicians of North Rhine between July 2019 and September 2020. To the best of our knowledge, the subspecialty “emergency medicine” is the only subspecialty in Germany where applicants are not required to have completed their training in a primary specialty and where entry to the subspecialty is possible for every physician after only 2 years of clinical experience.

However, our results show that those applying for board exam had a mean clinical experience of 5.9 (2.7) years despite 78% (85/112) of participants not having concluded training in their primary specialty. Most physicians had a background in anesthesiology, followed by internal medicine, surgery and general medicine. These findings are consistent with previous findings by Ilper et al. [9].

All but 4 participants had worked in intensive care with a mean experience of 13 (9.3) months and 75/112 participants (68%) were working in intensive care on a regular basis. Experience working in an emergency department was reported by only 50/112 participants (45%) with a mean experience of 9 (13.7) months. This study demonstrates that, despite the comparatively low requirements for board eligibility, those applying for the subspecialty usually had experience in treating critically ill patients that was considerably greater than the minimum requirement. In contrast, only 33/112 participants (30%) reported regularly treating pediatric patients, suggesting the need for a standardized minimum requirement.

### Airway procedures

Surgeons reported performing 89 (88.9) endotracheal intubations with direct laryngoscopy during their training, while internists performed 76.9 (64.9) intubations using direct laryngoscopy. A study by Konrad et al. [5] found a success rate of 90% after a mean of 57 intubations. However, after an initial steep increase in successful intubations, upon reaching an 80% success rate, the curve flattens considerably and larger numbers of intubations are required for modest further increases. Similar studies likewise found a number around 50 endotracheal intubations to correspond to a 90% success rate [6–9]. Bernhard et al. [10] found similar results and reported that the success rate stabilizes around 95% only after more than 150 attempts in an operating room environment. On the basis of these studies, the German Society for Anesthesiology and Intensive Care has issued a guideline for out-of-hospital airway management [14] recommending a benchmark of 100 endotracheal intubations for learning the procedure. Non-anesthesiologists did not meet the recommended number of procedures and the high standard deviations indicate that there are significant differences between those applying for board certification. Likewise, a minimum of 50 procedures is recommended for learning endotracheal intubation using video laryngoscopy [14] which non-anesthesiologists also did not achieve.

Despite the fact that the laryngeal tube is still the most available “alternative” airway, all participants of this study had little experience with the laryngeal tube. This may be attributable to generally low utilization of this device in hospitals when compared with prehospital care. Non-anesthesiologists used a laryngeal tube more frequently out-of-hospital than anesthesiologists. We hypothesize that this is due to most anesthesiologists having little experience using this device and therefore choosing more familiar measures for securing the airway. The higher prehospital experience of non-anesthesiologists with this device might therefore be an expression of lacking expertise in endotracheal intubation.

Mohr et al. reported an 86% success rate for correct placement of a laryngeal mask airway after an experience of 40 performed procedures [15]. Current guidelines [14] recommend a minimum of 45 performed procedures for learning the skill of securing the airway using supraglottic devices. Surgeons did meet this requirement using laryngeal-mask airways, while internists did not meet this requirement regardless of the device used. It should be emphasized that supraglottic devices should only be used in the prehospital field when caregivers have previously undergone proper in-hospital training, as these devices are prone to malpositioning in inexperienced hands.

Cricothyroidotomy was rarely performed among all participants—though anesthesiologists and surgeons reported higher than expected numbers, which may signal that this question was misunderstood. To the best of our knowledge no data describing procedural volume required for skill mastery is available. Some reference procedural volumes can only be derived from mannequin studies, although the transferability of these results is questionable—especially for airway management skills [16]. Wong et al. [17] reported that after five attempts 96% of participants were able to successfully perform cricothyroidotomy and success rates plateaued after five attempts. Based on this study and the rare opportunities to learn this skill in clinical practice, a minimum of five cricothyroidotomies on a skill simulator could be recommended. However, simulator training was not the subject of this study.

### Breathing procedures

Komatsu et al. [8] reported a success rate of 80% after a mean performance of 27 bag-valve-mask ventilations. However, this appears to be a relatively small number and previous studies have shown that bag-valve-mask ventilation can be associated with a high rate of serious complications [18]. Current guidelines therefore recommend a minimum of 100 performed bag-valve-mask ventilations to adequately learn the skill [14]. Although on average participants met the requirements, the high standard deviation indicates that some participants did not.

While anesthesiologists had significantly more experience in mechanical ventilation, the experience for non-invasive ventilation was similar between anesthesiologists and internists. Surgeons reported only 8.8 (18.5) performed non-invasive ventilations in the prehospital field and 7.3 (12.4) in-hospital. To the best of our knowledge, there are no recommendations available regarding a minimum number to have proficiency with these procedures. However, we assumed that the reported numbers indicate a lack of expertise in this skill for the surgical participants of this study.

### Circulation procedures

Prior work demonstrated that the success rate for intravenous access is 80% after 56 ± 43 performed procedures [9]. Establishing intravenous access can be uniquely challenging in the prehospital environment, necessitating greater experience and expertise. The participants of this study reported much higher numbers than those previously published to obtain proficiency.

Interestingly, non-anesthesiologists had more experience with placement of an intraosseous access—both in- and out-of-hospital. Intraosseous access is often used when intravenous access is unsuccessful; therefore, this finding may reflect a higher likelihood of success with intravenous access among anesthesiologists relative to non-anesthesiologists. There is no recommended minimum procedural number for learning how to establish intraosseous access, but studies have demonstrated that the procedure is easy to learn and training models can be utilized to learn successful needle placement [19].

Needle thoracostomy was rarely performed on a patient during training by all participants. In the prehospital setting, tension pneumothorax is a rare event in countries like Germany where gunshot or stab wounds are not common [20, 21]. Furthermore, in the hospital environment, it is more common to perform a tube thoracostomy, which is a more definitive treatment. Therefore, training has to utilize cadaver and other training models to learn this technique. To the best of our knowledge, there is no recommended number of procedures for mastering tube thoracostomy. Ball et al. [22] showed a complication rate of 28% for this procedure with the level of training not being a risk factor for complications. Davis et al. [23] defined a number of 10 thoracostomies to distinguish between “novices” and “experts”. Surgeons were the most experienced providers with 25.4 (31.3) performed procedures, while anesthesiologists performed 11.4 (17.4) tube thoracostomies and internists performed 7.2 (10.8) procedures. However, the high standard deviation indicates significant variability among individual participants.

Electrical cardioversion, external pacing and defibrillation were all more frequently performed by internists. There are no recommendations concerning training in these procedures in international guidelines on resuscitation [24]. However, surgeons in particular reported very little experience with these procedures.

Tourniquets were rarely used during training whether in- or out-of-hospital. This is consistent with a previous study we conducted regarding the experience of paramedic personnel [25]. Although the use of tourniquets is easy to learn, due to how rarely they are used in real-world settings, it may be necessary to provide additional practical training for prehospital emergency medicine trainees. Several studies have shown that tourniquets are often applied with too little pressure, and may occasionally cause increased bleeding due to venous stasis [26].

Pelvic binders were used more often than tourniquets, but overall were still used relatively infrequently. Much like the application of a tourniquet, the procedure is generally easy to learn, but may require focused education due to its relative rarity.

### Ultrasound procedures

Point-of-care ultrasound is increasingly used in prehospital medicine as ultrasound machines have become smaller and more portable. Prehospital emergency physicians therefore have to be familiar with ultrasound protocols like the Focused Assessment with Sonography in Trauma (FAST) exam. Ma et al. reported an accuracy of 86.1% after 12 months of training or a total of 35 FAST exams [27]. Gracias et al. reported that the learning curve flatten out between 30 and 100 FAST examinations [28]. Chisholm et al. reported an increased competency in focused transthoracic echocardiography after a 10 h course and more than 45 performed ultrasounds. The German Society for Ultrasound in Medicine (DEGUM) has issued a certificate in Emergency Ultrasound [30]. As part of this certificate, candidates must provide proof of a minimum of 25 supervised FAST exams and 80 supervised focused echocardiography exams. This provides some reference for interpreting the results of our study. Based on this reference, and given the findings of the current study, it has to be assumed that anesthesiologists as well as non-anesthesiologists are able to perform a sufficient FAST exam. However, anesthesiologists did not meet the recommended minimum of 80 supervised focused echocardiography exams.

### Limitations

The formal educational requirements for the subspecialty of prehospital emergency medicine do not incorporate a minimum number of performed procedures or require evidence of the number of performed procedures. Therefore, our data reflect participants’ estimates of the number of procedures they performed during training. Given the survey format, we cannot exclude the possibility of selection or non-response bias. However, with a response rate of 50.67%, the study would in general be considered representative. As the survey was conducted after passing the board exam, respondent anticipation of negative consequences is unlikely to have impacted response rate. As there are no formal requirements concerning procedural volume and participants of the study had just successfully passed the board exam, a self-disclosure bias is unlikely to be a source of relevant systematic error. Given a mean clinical experience of the respondents of 5.9 years, it can be assumed that there is some degree of recall-bias that has to be taken into account when interpreting the results of this study. However, especially for rarely performed procedures, it is likely that the participants were able to recall the number of performed procedures very accurately. In higher numbers—meaning that one cannot recall but only roughly estimate the number of performed procedures—the estimation will likely be less accurate. Independent of the specific procedure, learning curves usually flatten out with a higher number of performed procedures. Meaning that differences between higher procedural numbers usually should not represent marked differences in competency, and this limitation is therefore less relevant for answering our research question. Furthermore, the reported estimates are generally plausible and in accordance with previous study results [13, 31].

The consistently high standard deviation likely indicates large interindividual differences in the number of performed procedures. Given these limitations, these results should be interpreted with some caution.

## Conclusions

In our study we evaluated how often emergency physicians applying for board exam performed certain procedures that are considered critical manual skills in prehospital emergency medicine. We compared the results with existing recommendations and data on learning manual skills—where these were available.

Only anesthesiologist emergency physicians met the recommended minimal number of procedures for endotracheal intubation via direct laryngoscopy or video laryngoscopy. Surgeon emergency physicians had very little expertise with non-invasive ventilation, pacemaker therapy and electrical cardioversion. Anesthesiologist emergency physicians did not meet the recommended minimal number of performed focused echocardiography exams.

It would therefore be reasonable to incorporate a recommended minimal number of performed procedures for each skill into the formal educational requirements to be eligible for board certification in prehospital emergency medicine, as well as implement a mechanism for tracking these procedures. However, for many of the procedures we assessed in this study, there is insufficient data for clear minimum requirements necessitating more research to determine these.

## Declarations

For this article no studies with human participants or animals were performed by any of the authors. All studies mentioned were in accordance with the ethical standards indicated in each case.
